# Supplementary material for: Silicon Carbide-Gated Nanofluidic Membrane for Active Control of Electrokinetic Ionic Transport
Source: Membranes (Basel). 2021 Jul 15;11(7):535. doi: 10.3390/membranes11070535 (PMC8303522; doi:10.3390/membranes11070535)
Supplement: Supplementary file 1 [file membranes-11-00535-s001.zip › membranes-1285696-SI.pdf]

Supplementary Information

Silicon carbide gated nanofluidic membrane for active control of electrokinetic ionic transport

Antonia Silvestri [1,2], Nicola Di Trani [2], Giancarlo Canavese [3], Paolo Motto Ros [1], Leonardo Iannucci [3], Sabrina Grassini [3], Yu Wang [2], Xuewu Liu [2], Danilo Demarchi [1] and Alessandro Grattoni [2,4,5,*]

[1] Department of Electronics and Telecommunications, Polytechnic of Turin, 10129 Turin, Italy; antonia.silvestri@polito.it (A.S); paolo.mottoros@polito.it (P.M.R.); danilo.demarchi@polito.it (D.D)

[2] Department of Nanomedicine, Houston Methodist Research Institute, Houston, TX 77030, USA; nditrani@houstonmethodist.org (N.D.T.); xliu@houstonmethodist.org (X.L.); ywang2@houstonmethodist.org (Y.W.)

[3] Department of Applied Science and Technology, Polytechnic of Turin, 10129 Turin, Italy; giancarlo.canavese@polito.it(G.C.); leonardo.iannucci@polito.it (L.I.); sabrina.grassini@polito.it (S.G.)

[4] Department of Surgery, Houston Methodist Research Institute, Houston, TX 77030, USA;

[5] Department of Radiation Oncology, Houston Methodist Research Institute, Houston, TX 77030, USA;

* Correspondence: agrattoni@houstonmethodist.org (A.G.); Tel.: +1-(713)-441-7324 (A.G.)

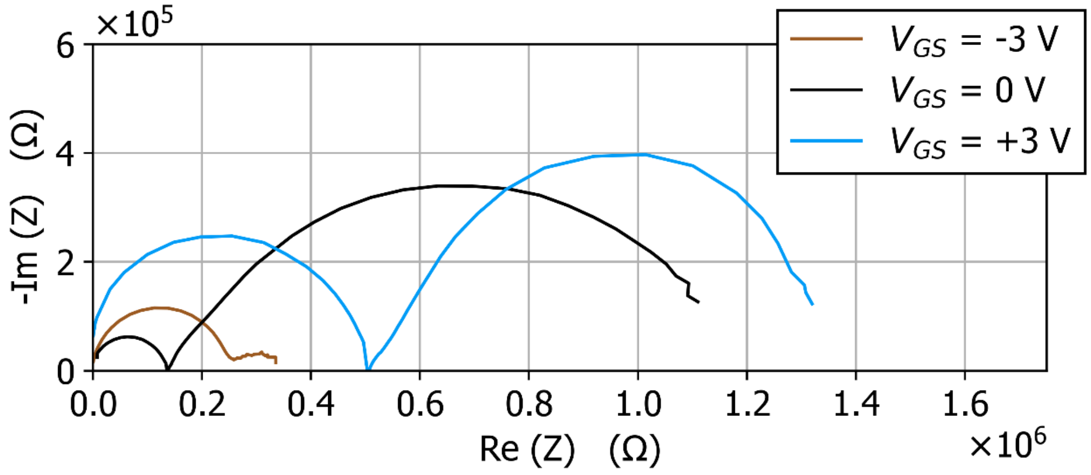

Figure S1. Impedance measurements presented as Nyquist plots performed on the a-SiC blank device recorded at different V_{GS}.
